# Delayed Healing of Tooth Extraction Sockets with Ramucirumab Use

**DOI:** 10.1155/2020/8881749

**Published:** 2020-09-30

**Authors:** Yosuke Iijima, Miki Yamada, Shunsuke Hino, Motohiko Sano, Takahiro Kaneko, Norio Horie

**Affiliations:** ^1^Department of Oral and Maxillofacial Surgery, Saitama Medical Center, Saitama Medical University, Saitama, Japan; ^2^Division of Applied Pharmaceutical Education and Research Hoshi University, Tokyo, Japan

## Abstract

**Objective:**

An angiogenesis inhibitor can cause medication-related osteonecrosis of the jaw (MRONJ). To our knowledge, there has been no report that an angiogenesis inhibitor causes delayed healing of tooth extraction socket. Here, we describe a case of delayed healing of tooth extraction sockets associated with an angiogenesis inhibitor, ramucirumab, which showed characteristics similar to MRONJ.

**Materials and Methods:**

A 76-year-old male patient, who was diagnosed with gastric cancer with liver metastasis, received tooth extraction twice during continuous chemotherapy comprising paclitaxel and ramucirumab.

**Results:**

The first extraction was performed 30 days after ramucirumab discontinuation without complication. The second extraction was conducted without ramucirumab discontinuation. Although tooth socket healing was finally achieved, it took about 150 days. *Discussion*. This case was considered to be delayed healing of dry sockets rather than MRONJ due to ramucirumab. Dentists and oral surgeons need to be aware that angiogenesis inhibitors can cause not only MRONJ but also dry sockets after tooth extraction.

## 1. Introduction

Many new cancer chemotherapeutic agents have been recently developed and administered. Among chemotherapeutic agents, angiogenesis inhibitors reduce or slow cancer progression by blocking the nutritional supply that the tumor requires. Ramucirumab, which was relatively newly approved by the United States Food and Drug Administration, binds to the extracellular domain of vascular endothelial growth factor-2 (VEGF-2) with high affinity and selectivity and blocks the binding of multiple VEGF ligands (VEGF-A, VEGF-C, and VEGF-D) to VEGFR-2 [1, 2]. Clinically, angiogenesis inhibitors are used alone or in combination with other chemotherapeutic agents. Ramucirumab has been used in second-line treatment of cancers such as gastric cancer, nonsmall cell lung cancer, and colorectal cancer [2].

Chemotherapeutic agents cause various adverse events, and major adverse events of angiogenesis inhibitors are hypertension, vomiting, neutropenia, and anemia [3]. Angiogenesis inhibitors can also interfere with wound healing, which is caused by blocking of vasodilation, increased vascular permeability and angiogenesis, and complication of wound healing was found in 0.5% of patients treated with ramucirumab [2, 4].

Medication-related osteonecrosis of the jaw (MRONJ) has been identified as a common oral adverse event of chemotherapy [5]. MRONJ is principally caused by bone-modifying agents (BMAs) including bisphosphonates and denosumab, which inhibit bone resorption, and MRONJ also occurs upon taking angiogenesis inhibitors without the use of BMAs [5, 6]. Invasive dental surgery, such as tooth extraction, is the predisposing factor of MRONJ.

To date, there has been no report of delayed healing of a tooth extraction socket complicated by MRONJ during ramucirumab use. In this article, the authors report on two tooth extractions in a patient treated with ramucirumab. The first tooth extractions occurred 30 days after ramucirumab discontinuation and the sockets healed well. The second extractions were performed without ramucirumab cessation and severe contact pain of the socket quickly developed. Although healing was finally possible, it took about 150 days for the socket to heal completely. From a comprehensive perspective, these findings suggested that the second extraction sockets might be caused by delayed dry socket healing (alveolar otitis) rather than MRONJ.

## 2. Case Report

**Figure 1:**
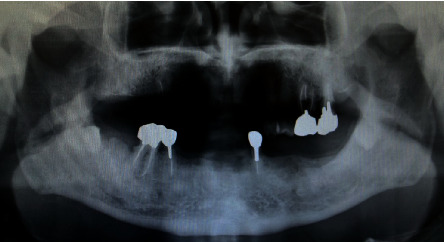
Photoradiograph showing the patient's teeth before the first extractions.

**Figure 2:**
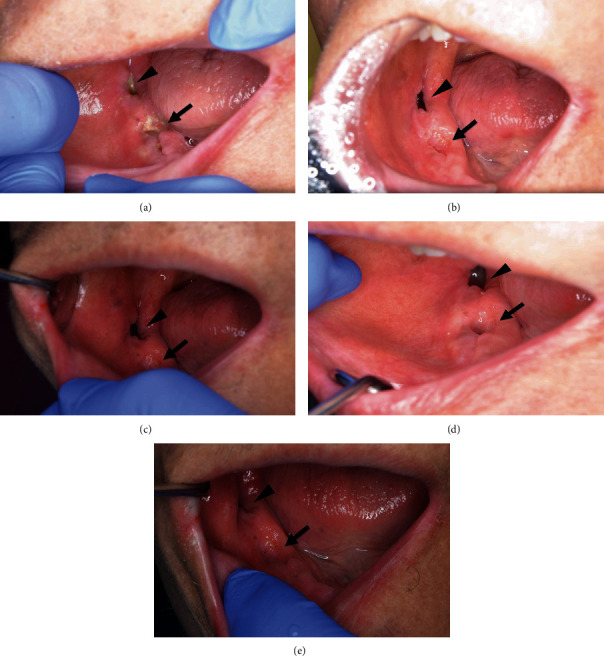
Photographs showing the healing process of the sockets. (a) Bone exposure was noted 23 days after the extractions in each socket (arrow, sockets of the first molar and second premolar; arrowhead, socket of the third molar). (b) Sixty-one days after the extractions, sockets of the first molar and second premolar were mostly covered with soft tissue (arrow), whereas the socket of the third molar was not covered (arrowhead). (c) Seventy-five days after the extractions, sockets of the first molar and second premolar were almost completely covered with soft tissue (arrow), but the socket of the third molar was only slightly covered (arrowhead). (d) One hundred and seventeen days after extractions, sockets of the first molar and second premolar were completely covered with soft tissue (arrow). However, the socket of the third molar was only partially covered (arrowhead). (e). One hundred and thirty days after extraction, the socket of the third molar was almost completely covered with soft tissue (arrowhead).

In July 2018, a 76-year-old man was referred to the oral surgery clinic from the gastroenterology and hepatology clinic for dental caries treatment. In August 2016, the patient was diagnosed with gastric cancer with multiple liver metastases and lymph node metastases. The patient began chemotherapy comprising cisplatin and tegafur/gimeracil/oteracil. In February 2017, the lymph node metastases had shrunk and the patient underwent surgery for gastric cancer. Subsequently, beginning in June 2017, he started chemotherapy comprising paclitaxel (100 mg) and ramucirumab (310 mg) as second-line treatment. Paclitaxel was given weekly, and ramucirumab was given every 2 weeks. The patient was also prescribed concomitant antihypertensive and diuretic medications. In July 2018, there was no evidence of recurrence of liver metastasis by positron emission tomography. Furthermore, the patient desired to treat dental caries and stop chemotherapy; thus, chemotherapy was discontinued. Thirty days after the last dose of ramucirumab, the right maxillary central incisor, right maxillary second premolar, left maxillary first and second molars, and left mandibular lateral incisor were extracted (Figure 1). The postextraction course was uneventful with good healing of tooth extraction sockets. In November 2018, computed tomography showed recurrence of liver metastasis and the patient restarted chemotherapy with paclitaxel and ramucirumab (same dose as before). In January 2019, the patient experienced repeat pericoronitis in the right mandibular third molar and eating difficulties. Thus, the right mandibular third molar and right mandibular first molars and second premolar, which were difficult to treat conservatively, were extracted in March 2019 without ramucirumab discontinuation after discussion between the patient and the chemotherapy team. The extractions were performed 8 days after ramucirumab administration, taking into consideration the half-life of ramucirumab (8 days) and the timing of the next administration of ramucirumab. The third molar, which was an impacted tooth, was extracted with elevation of the mucoperiosteal flap and bone removal. After the tooth extractions, the patient received amoxicillin (750 mg) for 7 days, and acetaminophen (400 mg) was given as an analgesic. Seven days after the extractions, the patient felt strong contact pain in the sockets. He had no other symptoms that suggested the spread of inflammation. Dry sockets were strongly suggested, and the analgesic was continued. Paclitaxel and ramucirumab were restarted according to the chemotherapy regimen. Twenty-three days after the extractions, the patient stated that he was still in severe pain but the pain was better than before (Figure 2(a)). Subsequently, paclitaxel and ramucirumab were continued. Twenty-five days after the extractions, the lower denture was extended to cover the extraction socket to decrease the contact pain. Forty-seven days after the extractions, the pain was moderately diminished, but soft tissue did not completely cover the extraction sockets. Sixty-one days after the extractions, the pain was considerably diminished, and soft tissue mostly covered the extraction sockets of the first molar and second premolar, but not that of the third molar (Figure 2(b)). This was probably because the third molar extraction was more invasive with bone removal. Seventy-five days after the extractions, the socket of the third molar was slightly covered with soft tissue (Figure 2(c)). One hundred and three days after the extractions, the socket of the third molar was still not completely covered with soft tissue. One hundred and seventeen days after the extractions, the socket of the third molar was partially covered with soft tissue. There was almost no contact pain. By comparison, the extraction sockets of the first molar and second premolar were completely covered with soft tissue (Figure 2(d)). One hundred and thirty days after the extractions, the extraction socket of the third molar was almost completed covered with soft tissue with almost no pain (Figure 2(e)). On the same day, the final doses of paclitaxel and ramucirumab were given because the patient's liver metastasis subsequently progressed. Third-line treatment with nivolumab (240 mg) was started 158 days after the tooth extractions, and the patient reported no pain in the sockets at this time. One hundred and eighty-four days after tooth extractions, the patient continued to receive nivolumab, and complication of the socket such as bone exposure was not observed.

## 3. Discussion

Dental treatment of patients during chemotherapy, especially surgical intervention, is recommended prior to chemotherapy and, when absolutely necessary, during chemotherapy intervals. However, chemotherapy is seldom needed once, commonly requiring repeated administrations with a long duration, and the interval between chemotherapy cycles may be too short for adequate dental treatment. Furthermore, depending on the patient's condition, it may be necessary to provide dental treatment without considering chemotherapy withdrawal and interval periods.

MRONJ is defined as the presence of exposed bone or bone that can be probed through an intraoral or extraoral fistula in the maxillofacial region, which is present for more than 8 weeks and without a history of radiation therapy to the jaws [5]. In this case, delayed healing of the second extraction socket occurred with bone exposure of more than 8 weeks, and the patient had no history of radiation therapy to the jaws or metastatic disease to the jaws. Based on these findings, the patient would typically be considered to have stage 2 MRONJ. However, the authors were concerned that this case differed from the typical MRONJ clinical course.

As with MRONJ, dry socket is a common complication after tooth extraction, wherein the socket is not covered with soft tissue. Fibrinolysis of blood clots has been considered the main cause of dry socket. Although the mechanism is unclear, bacterial involvement has been suggested [7]. The socket is partly covered or totally devoid of blood clots with exposed, rough, painful bone. Considering the action of angiogenesis inhibitors, it is quite possible that these agents cause dry sockets. Dry sockets are characterized by severe and persistent pain, which lasts till the exposed (and nonvital) bone is either eventually covered by granulation tissue or is separated from the underlying bone and sequestrated. This process can take more than 4 weeks.

In this case, severe pain occurred soon after extraction. In a case of dry socket, it is usually sharp pain. There may be local lymphadenitis, and a few untreated cases of dry socket may progress to infection that spreads through the bone marrow (osteomyelitis) [8]. Pain is improved by covering the wound, as in this case. However, in MRONJ, severe pain rarely occurs immediately after extraction, inflammation gradually increases, and antibiotic therapy is effective to improve the patient's condition. In this case, there was no pus discharge from the extraction sockets, and the extraction sockets healed without continuous antibiotic therapy, although it took a long time. These results suggested that ramucirumab may cause relatively long-delayed healing of dry sockets, and patients may exhibit symptoms similar to MRONJ or meet MRONJ diagnostic criteria. In comparison to bevacizumab, which binds to VEGF-A and is the first clinically used angiogenesis inhibitor, ramucirumab blocks the binding of multiple VEGF ligands. This difference in ligand blocking may be related to the formation of a delayed healing dry socket.

Pimolbutr et al. reported 35 cases of MRONJ associated with angiogenesis inhibitors other than ramucirumab [6]. The authors described that MRONJ caused by angiogenesis inhibitors had superior healing rates than MRONJ caused by BMAs. Thus, there might be a qualitative difference in bone modification effects between MRONJ caused by BMAs and MRONJ caused by angiogenesis inhibitors.

If delayed socket healing is caused by a common dry socket rather than MRONJ, then treatment for a dry socket is necessary. Good oral hygiene is necessary not only to keep the socket clean but also to prevent adverse events associated with concomitant use of chemotherapeutic agents. Good oral hygiene is also essential for MRONJ treatment. In addition, analgesics are effective to manage postoperative pain in dry socket. Antibiotics may reduce infection when used as surgical prophylaxis, but postoperative administration is not recommended [8, 9]. Irrigation with warm saline to remove the debris, chlorhexidine mouthwash, and dressings to cover the exposed bone are effective. In this case, it was possible to alleviate the patient's oral pain by extending the denture to cover the extraction sockets.

In conclusion, this case was considered to be delayed dry socket healing rather than MRONJ caused by ramucirumab. If a patient has a dry socket, the dry socket treatment is necessary. Dentists and oral surgeons need to be aware that angiogenesis inhibitors can cause not only MRONJ but also dry sockets after tooth extractions that are very slow to heal.
